# Establishing and Managing a Global Student Network

**DOI:** 10.1371/journal.pcbi.1003920

**Published:** 2014-10-30

**Authors:** Avinash Shanmugam, Geoff Macintyre

**Affiliations:** 1 Department of Computational Medicine and Bioinformatics, University of Michigan, Ann Arbor, Michigan, United States of America; 2 The Centre for Neural Engineering, University of Melbourne, Parkville, Victoria, Australia; National Cancer Institute, United States of America and Tel Aviv University, Israel

## Overview

The Regional Student Groups (RSGs) program is a network of student groups affiliated with the International Society of Computational Biology Student Council. While each RSG is encouraged to act independently and address the local needs of its regional student membership, a significant amount of effort is also invested in coordinating affiliation of these groups with the international student body, which provides long-term direction and facilitates communication between groups. Participating in a global student network provides students with an opportunity to network and connect with others around the globe. By sharing experiences within the network, students gain cultural insights and awareness of regional differences in scientific research and industry. In this article, we provide an overview of the tasks involved in setting-up and managing this global student network for bioinformatics. We also highlight the benefits a global student network offers, in the hope that other fields can use this to create their own global student network.

## The Regional Student Group Network

From a humble beginning of four RSGs six years ago, the RSG network has grown to encompass close to 20 different RSGs spread across the globe [1]. With the growth of the network, made up entirely of volunteers, it became necessary to have a dedicated volunteer to oversee the network's operation. A RSG chair was appointed in 2008 to manage the network and communicate all RSG happenings to the Student Council leadership. However, with even greater expansion of the network over the last year, the responsibilities became too much for one volunteer. Therefore, the responsibilities were split over three new regional vice-chairs who work with the RSG chair to oversee the operation of RSGs in Europe, Asia, and Africa. Together this committee ensures that each RSG is functioning well, facilitates communication between RSGs, and manages all RSG registrations and renewals. The vice-chairs touch base regularly with RSGs, and together the RSG committee develops strategies and initiatives that benefit all RSGs in the network. It is the job of this committee to anticipate changes in the network (especially if it continues to expand) and put measures in place so that the network grows and remains sustainable into the future.

## Why Build a Network?

Improved online communication and networking means that science is being carried out at an increasingly international level. While a RSG alone is great for addressing the needs of local students, an RSG as part of a global network facilitates sharing of ideas, improves efficiency of group operations through sharing of operational procedures, and provides student members with valuable international networking experience. Once up and running, a global network takes surprisingly minimal effort to maintain, providing the workload is shared, and the benefits are enormous [2].

## Establishing a Network

To build a dynamic network, there need to be multiple student groups in different regions around the globe with a shared interest. See the “Ten Simple Rules for Starting a Regional Student Group” [3] article on how to set up a group. In most cases, however, these groups do not need to be started from scratch. There will inevitably be a number of groups of students that meet regularly, scattered around the globe, which could benefit from being connected through a larger network. The effort lies in identifying and unifying these groups. The best way to identify if multiple groups are already in existence is to quiz fellow students at international conferences and meetings. In our experience, the Student Council continues to identify established student groups in the field of computational biology that can benefit from being part of the global RSG network. If, however, the network needs to be created from scratch, we recommend starting with a number of “seed” groups. Two or three groups in different locations around the world will provide a great start to forge a new network.

In either case, it is important from the outset to have a mechanism to unify the groups. Ideally, affiliation with a larger, established, non-student parent body is a great way to attract groups to be part of the network (in our case, this is the International Society for Computational Biology). The parent body can provide advice (and, potentially, mechanisms for funding) to the groups in the network, and affiliation with this body can provide an element of recognition. As well as a parent body, it is important to have a committee overseeing the operation of all groups (this could initially include some of the heads of each group). It is crucial to make sure this committee pays careful attention to the operation of each group. The success of the network will be judged on how these groups perform in the early days. This will be a learning stage, with many unanticipated issues arising. It is a good idea to document lessons learned, so that new groups (and future committees) can learn from the experience of others. It is also good to identify any rules and regulations that may help in making the network work more efficiently (usually derived from the lessons learnt!).

Basic infrastructure needs to be put in place to ensure the network is visible and all members in the network can communicate successfully. For example, a dedicated email address, website, and online resources must be made available to all network RSGs. The International Society for Computational Biology (ISCB) Student Council RSG network uses an online project management tool to facilitate communication and document interactions with great success. If all goes well, the groups as part of the network will start to see the benefits of being connected. Once this has been achieved, it is time to start expanding the network.

## Expanding the Network

In the beginning of the ISCB Student Council RSG network, expansion of the network was done mainly by showcasing the activities and achievements of existing RSGs during various conferences and inviting interested students to begin new RSGs in their country (or region) [3]. The Student Council has also had success in promoting the RSG network on its website and inviting applications from interested students over the internet.

There is usually a three month time frame from when a group of students express interest in starting up an RSG to actually getting everything in place and the RSG being officially recognized. During this time the applicants get an idea of the time commitment involved in running an RSG. This process ensures that all recognized RSGs have an enthusiastic and committed leadership. A RSG with a good leadership will inevitably contribute positively to the network. Ultimately, expansion of the network relies on good advertising of the successes of the groups currently in the network.

## Managing the Network

It is important that the RSG committee be diligent in their management of the network. This includes simple practices such as being prompt in responding to communications. Not receiving timely responses can negatively impact the motivation of members of the network. Here we outline the three most important areas to successful operation of the ISCB-SC RSG network: communication between RSGs, creating value for the RSGs, and accountability.

### Communication between RSGs

The most important asset to the RSG network is successful communication. There are common themes that run through the activities that each RSG carries out; therefore, it is of benefit for RSGs to communicate and learn from each other. For this reason, encouraging communication within the RSGs is a priority. The major means of communication between the RSGs in the ISCB Student Council is a project management website used by the Student Council set up specifically for RSG-related discussions. RSGs are encouraged to share news of events and initiatives that they execute and solicit ideas for any future projects, if needed, in this forum. We recommend adopting something similar if creating a network.

In addition, RSGs from the same part of the world are also encouraged to interact more closely. Towards this end, the RSG committee added regional vice-chairs for Europe, Asia-Pacific, and Africa. The vice-chairs organize periodic Skype calls with RSGs from their region as a way of increasing communication and cooperation within the region. This is another great way of getting RSGs working together.

Another important avenue of communication between RSGs is personal meetings on the sidelines of conferences. Alongside the organic spread of RSGs around the world, certain conferences have come to form natural meeting spots for certain regions, such as the European Conference on Computational Biology (ECCB) for Europe, the International Society for Computational Biology–Africa conference (ISCB-Africa) for Africa, the Asian Young Researchers Conference on Computational and Omics Biology (AYRCOB) and International Conference on Bioinformatics (InCoB) for the Asia-Pacific region. Having identified these trends, new RSGs in those regions are encouraged to attend these conferences when possible. Being part of a global student network is usually seen positively when travel fellowships are selected, thus making it easier for RSG committee members to attend.

### Creating value for RSGs

To maintain a successful network, it is important to strive to provide value for the RSGs that are involved in the network. One way this is done through the Student Council is to provide a RSG funding program. The RSG funding program is designed to support RSGs in organizing various events. This funding support allows RSGs without other sources of funding to organize events and quite often acts as seed money, allowing them to attract more funds. Presently the Student Council funds four to five RSG events per year, with each RSG having the opportunity to apply for up to US$400 in funding. RSGs can also decide to collaborate with each other and send in a combined funding proposal, which can be allocated more funds.

Other avenues of value addition to the RSG network can be as simple as providing web hosting infrastructure, or expertise with fundraising efforts. A committed effort to add value to RSGs being part of the network will go a long way in creating a dynamic and vibrant community.

### Accountability

Affiliation of a RSG with a global network demands a degree of responsibility. It is the responsibility of each RSG to operate in the best manner they can and create value for their members. If a RSG in the network is not able to serve its student community actively, it can affect the morale of other groups in the network. It is therefore good to have some degree of accountability for affiliation with the network. While this cannot be done with an iron fist in a network of all volunteers, a few practices can be put in place that encourage RSGs to perform at their best. The ISCB Student Council uses regular Skype meetings and an annual renewal of affiliation to accomplish this. Each RSG must submit a short annual report documenting their achievements over the past year and goals for the next with a detailed plan. This has helped RSGs stay on track and assisted in identifying when an RSG can no longer operate. Another great way to keep RSGs motivated is to make RSG progress public across the network. Seeing the performance of active and vibrant RSGs can be a useful motivator for other RSGs to improve their performance and borrow successful ideas from the network.

## Conclusions

By providing insight into the operations of the ISCB Student Council Regional Student Group network, we hope to inspire the creation of new global student networks in other fields. Each member of our global network can vouch for its benefits, and an amazing number of achievements have been made to date (some highlighted in the article series of which this article is part). Furthermore, being part of a global network naturally leads to bigger and better opportunities. Four of the five leaders in the ISCB Student Council Executive team became involved in the Student Council through the RSG network.

The tips and tricks for starting a global network provided in this article are a great starting point to get a network off the ground. So what are you waiting for?

**About the Authors**

The authors have been involved in organizing various events for Regional Student Groups and the ISCB Student Council. **Avinash Shanmugam** was president of RSG-India (2008–2009), the RSG Committee chair (2009–2012), and is currently the Student Council representative to the ISCB Board of Directors (since January 2013). **Geoff Macintyre** served as chair of the ISCB Student Council (January 2011–January 2013) and was co-founder and president of RSG-Australia (2009–2010).
